# Interdisciplinary CardioVascular and Thoracic Surgery—2023 Reviewers

**DOI:** 10.1093/icvts/ivae035

**Published:** 2024-03-25

On behalf of EACTS and EBCP, the Editor-in-Chief wishes to thank the following colleagues who have voluntarily given their valued time and effort to reviewing papers for ICVTS in 2023.

Seyed Hossein Aalaei Andabili

Mathias H. Aazami

Cipriano Abad

Udo Abah

Ahmed Abbas

Jonathan R. Abbas

Mohammed Abbas

Sharjeel Abbas

Sherif Osama Ahmed Abbas

Kyomars Abbasi

Mohammed Nabil Abd AlJawad

Mohamad Hafizan Abd Hadi

Hassane Abdallah

Mohamed Abdel Bary

Ahmed Abdel Razek

Moslem Abdelghafar

Khaled Abdelhady

Osama Abass Abdelhameed

Mohammed Gouda Abdellatif

Taher AbdelMoneim

Mahmoud Hassan Abdelnabi

Michael Abdelnoor

Etienne Abdelnour-Berchtold

Amir Abdelsayed

Amr Abdelmonem Abdelwahab

Aida Abdelwahed

Ashiq Abdul Khader

Shinji Abe

Sosei Abe

Tomonobu Abe

Yukio Abe

Meredith Abel

Karen Beatrix Abeln

Hannes Abfalterer

Moussa Abi Ghanem

Said Abisi

Djamila Abjigitova

Khaldoon Abo Dakka

Moustafa Farouk Aboollo

Youssef Fady Abouelela

Armando Abreu

Vijoleta Abromaitiene

Firas Abu Akar

Walid Abu Arab

Yasir Abu Omar

Burçin Abud

Ashraf Abugroun

Masood Abu-Halima

Arkin Acar

Roberto Accinelli

Geeta Acharya

Metesh Nalin Acharya

Paul Achouh

Burak Acikgoz

Stefan Acosta

Guglielmo Mario Actis Dato

Niv Ad

Iki Adachi

Hiroyuki Adachi

Uyanga Adam

Adem Adar

Alfredo Addeo

Srilakshmi M. Adhyapak

Rajeshwara Krishna Prasad Adluri

Bouke Adriaans

Prasad S. Adusumilli

Alexander V. Afanasyev

Jonathan Afoke

E. Andreas Agathos

Pankaj Aggarwal

Constantina Aggeli

Andrea Agostinelli

Apostolos Agrafiotis

Hitesh Agrawal

Deepi Goyal Agrawal

Ankit Agrawal

Heba Aguib

Rio J. Aguilar

John Agzarian

Seyedhossein Aharinejad

Anders Ahlsson

Seyed Hossein Ahmadi Tafti

Kamran Ahmadov

Adnan Ahmed

Ahmed Ahmed

Elnazeer Osman Ahmed

Raheel Ahmed

Zakarya Ahmed

Hyuk Ahn

Seha Ahn

Diana Aicher

Clemens Aigner

Nadia Aissaoui

Jenni Aittokallio

Nina Ajmone Marsan

Kentaro Akabane

Olutosin Akanni

Serdar Akansel

Ahmet Ruchan Akar

Erdyanto Akbar

Ferdi Akca

Rami Akhrass

Zaki Akhtar

Noori Akhtar-Danesh

Payam Akhyari

Berdel Akmaz

Enoch F. Akowuah

Yunus Aksoy

Mohammed Al Aboud

Ousama Al Habash

Zohair Al Halees

Charles Al Zreibi

Mohammad Abdelrahman Aladaileh

Mohamed Al-Aloul

Henna Maria Ala-Seppälä

Ilker Alat

Nawwar Al-Attar

Turki B. Albacker

Alexander Albert

Johannes Maximilian Albes

Alya Alblooshi

Ahmed Abdul Wahab Al-Bulushi

Maria Inmaculada Alcalde Mayayo

Jorge Alcocer

Ibrahim Aldobayyan

Prince Andrew Alebna

Ivan Aleksic

Loukia Alexopoulou-Prounia

Cem H. Alhan

Fatima Alharmoodi

Heba Ali

Jason M. Ali

Menna Ali

Stephen Ali

Sulafa Khalid Mohamed Ali

Umar S. Ali

Marco Alifano

Rahi Alipour Symakani

Ali Aljalloud

Hesham Mostafa Alkady

Hani N. Alkattan

Khaled M. Al-Kattan

Mohamad Alkhouli

Ahmad Alli

Fabiano Almeida

Saleh Alnasser

Mohammed Al-Nejar

Obada Ahmad Alqudah

Hussain AlShimali

Bahaaldin Alsoufi

Peter Alston

Salah Eldien Altarabsheh

Antonio Alvarez

Julián Álvarez Escudero

Adelaide Alves

Andrea Amabile

Ignacio J. Amat-Santos

Efstathios Ambazidis

Shirish G. Ambekar

Paurush Ambesh

Vincenzo Ambrogi

Marcello Carlo Ambrogi

Márta Ambrus

Angela Amigoni

Armin Amirian

Ganesh Kumar K. Ammannaya

Luca Ampollini

Sergei Amunts

JianXiong An

Kang An

Kevin Ruixuan An

Xiang-Guang An

Xiuping An

Olga Gregorios Ananiadou

Mahesh Anantha Narayanan

Kyriakos Anastasiadis

Nicholas D. Andersen

Robert H. Anderson

Marco Andolfi

Rafael Andrade

Dario Andrade Fierro

Terézia B. Andrási

Martin Andreas

Claudio Andreetti

Maria Jose Andres-Prado

David T. Andrews

Eleni-Rosalina Andrinopoulou

Udo Christian Anegg

Gianni D. Angelini

Philipp Angleitner

Dimitrios C. Angouras

Marco Anile

Saipavan Anne

Alfredo Annicchiarico

Andrea Daniele Annoni

Chris Anthony

Phillip Antippa

Carlo Antona

Filippo Antonacci

Miha Antonic

Athanasios Antoniou

George Antoniou

Shabana Anwar

Denish Apparau

Mohammad Arab

Muhammad Refaat Arab

Bardia Arabkhani

Amr A. Arafat

Shusuke Arai

Yoko Arakawa

Pedro J. Aranda

Ramón Aranda-Domene

Raghuram Arani

Arunkumar A. Arasappa

Nurcan Arat

Claudia Arenz

Giuseppe Aresu

Ricardo Jose Argueta Cruz

Mostafa Ariafar

Olgun Kadir Aribaş

Priyadharshanan Ariyaratnam

Albert Ariza Sole

Francesco Arlati

Matthias Arlt

Giovanna Armani

George J. Arnaoutakis

Zsuzsanna Arnold

Rakesh Christopher Arora

Mani Arsalan

Gokhan Arslan

Gökhan Arslanhan

Panagiotis Artemiou

Krishnasamy Arunkumar

Tohru Asai

Mitsuru Asano

Ammar Asban

Guido Ascione

Boulos Asfour

Fatih Halil Asgun

Amr Ashry

Emre Kartal Aslanger

Luis Asmarats

Melyana Asmuni

Sanjay S. Asopa

Omid Assar

Anna Kathrin Assmann

Eduardo Astrosa Martin

Ryo Ataka

Thanos Athanasiou

Rony Atoui

Jun Atsumi

Saina Attaran

Ahmed Attia

Rizwan Q. Attia

Tim Attmann

Waleed Aty

Emily Au

Vincent Auffret

John Augoustides

Daniel Auinger

Erle H. Austin

Nicholas Austine

Rüdiger Autschbach

Sarit Aviel-Ronen

Mark Nelson Awori

Hüseyin Ayhan

Koga Ayumi

Luis Filipe Azenha Figueiredo

Mohammad Babgi

Mahesh B. Babu

Frank A. Baciewicz Jr.

Carl L. Backer

Vinay Badhwar

Vishal Badiger

Catalin Constantin Badiu

Gabor Bagameri

Leila Baghernajad Salehi

Yunpeng Bai

Nikolaos G. Baikoussis

Manjit Bains

Shen Bairu

Pietro Bajona

Shiraslan Bakhshaliyev

Farhad Bakhtiary

Ihsan Bakir

Robert Balan

Joan Balcells

Husam Balkhy

Felix Balzer

Oluwaseun Adebayo Omotayo Bamodu

Ko Bando

Toru Bando

Saraansh Bansal

Yi Bao

Ralitsa Baranowski

Marco Barbanti

Annalisa Barbarossa

Cristina Barbero

Clay Matthew Barbin

Jérémy Bardet

Fabio Barili

Fabrice Barlesi

Giuseppe Barletta

Olivier Baron

Carlo Bartoli

Jean-Marc Baste

Timothy James Batchelor

Hasan Fevzi Batirel

Robin Antoine Baudouin

André Bauer

Timm Bauer

Kathrin Bäumler

Nicolai Christian Bayer

Nicholas Bayfield

Christopher David Bayliss

Yusuf Bayrak

Gwyn William Beattie

Thomas M. Beaver

Craig Beavers

J. F. Matthias Bechtel

Erik Beckmann

Eric Louis-Robert Bedard

Benoît Bédat

Benedetta Bedetti

Danielle Bedin

Andres Beiras-Fernandez

Elizabeth Belcher

Jose Belda-Sanchis

Reda Belhaj Soulami

Jared P. Beller

Emre Belli

Jocelyn Bellier

Irene Bello

Hannah Rosemary Bellsham-Revell

Sabrina Ben Ben-Ahmed

Umberto Benedetto

Leila Louise Benhassen

Martina Benker

James Bentham

Stefano Benussi

Bogdan Berbescu

Denis A. Berdajs

Mikolaj Berezowski

Lennart Bergfeldt

Pascal Berna

Melissa Bersanelli

Alessandro Bertani

Luca Bertoglio

Pietro Bertoglio

Luca Bertolaccini

Morris Beshay

Léa Betser

Muhammet Ali Beyoglu

Branislav Bezak

Suneet Bhansali

Amit Bhargava

Fausto Biancari

Giacomo Bianchi

Maria Fernanda Biancolini

Benoit Jacques Bibas

Frederike Bieling

Rocco Bilancia

Andrea Bille

Giuseppe Biondi-Zoccai

Arvind Bishnoi

Gianluigi Bisleri

Mohamad Nidal Bittar

Henrik Bjursten

Sabine Bleiziffer

Marc Boada

Simon C. Body

Dietmar Boethig

Lyubomyr Bohuta

Ciprian Bolca

Servet Bölükbas

Nikolaos Bonaros

Johannes Bonatti

Stefano Bongiolatti

Andreas A. Böning

Kasper Bonnesen

Florina Borca

Jochen Börgermann

Mimi Rosealie Borrelli

Amal K. Bose

Wolfgang Bothe

Petre Vlah-Horea Botianu

Brandi Bottiger

Tomaso Bottio

Fairuz Boujibar

Anas Boulemden

Thierry Bourguignon

George Johannes Brandon Bravo Bruinsma

Andrew Brazier

Christian Pierre Brizard

Christoph Brochhausen

Alessandro Brunelli

Vito Domenico Bruno

Giuseppe Bruschi

Kamil Bujak

Marianna Buonocore

Hans Ulrich Burger

Nicholas Burris

Jawad Haider Butt

Lucio Cagini

Ufuk Cagirici

Jing-Sheng Cai

Teng Cai

Garrett Matthew Cail

Antonio Maria Calafiore

Duke Edward Cameron

Alessio Campisi

Christopher Cao

Qi Cao

Massimo Caputo

Giovanni Luca Carboni

Giuseppe Cardillo

Manuel Carnero-Alcazar

Matthew James Carr

Yolanda Carrascal

Thierry Carrel

Angelo Carretta

Raymond Cartier

Monica Casiraghi

Filip P. Casselman

Patrizio Castelli

Javier G. Castillo

Thierry Caus

Francesco Cavallin

Elif Ijlal Cekirdekci

Robert J. Cerfolio

Alfredo Giuseppe Cerillo

Nikola Cesarovic

Laurens J. Ceulemans

David J. Chambers

Stephanie Chan

Feng-Cheng Chang

Jen-Ping Chang

Yin-Kai Chao

Ahmed Mehdi Chaoui

Andrew C. Chatzis

Alexander Vladimirovich Chausov

Bing-Ying Chen

Hao Chen

Huang Chen

Ping Chen

Robert Jeenchen Chen

Cai Cheng

Yeung-Leung Cheng

Ilaria Chirichilli

Walter Randolph Chitwood Jr.

Sian Catherine Chivers

Hyun Jin Cho

Jong Bum Choi

Wei-Han Chou

Yi-Pin Chou

Shiv Kumar Choudhary

Torsten Christ

Rasmus Christensen

Michael W. A. Chu

Xiao Chu

Jennifer Chia-Ying Chung

Sertac M. Cicek

Ángel Cilleruelo

Hüseyi̇n Ulaş Çınar

Paola Ciriaco

Joseph Brian Clark

Julie Cleuziou

Andrea Colli

Giuseppe Comentale

Miguel Congregado

Lenard Conradi

Massimo Conti

Monica Contino

Hannah Copeland

Dragan Copic

Joseph S. Coselli

Joseph Costa

Victor Costache

Elvio Covino

Garrett N. Coyan

Gunseli Cubukcuoglu Deniz

Weixue Cui

Martin Czerny

Gibran Ribeiro da Rocha

Gry Dahle

Magnus Dalén

Giuseppe D’Ancona

Antonio D’Andrilli

John Dark

Laleng Mawia Darlong

Joao-Carlos Das-Neves-Pereira

Hiroshi Date

Piroze Davierwala

Fabio Davoli

Nieves De Antonio Antón

Julie De Backer

Jose Ribas Milanez De Campos

Jafar De Cassem

Vinicio de Jesus Perez

Laurent De Kerchove

Mercedes de la Torre Bravos

Vincent De Pauw

Raffaele De Simone

Andrea De Vico

Georges Decker

Aikaterini Dedeilia

Benedetto Del Forno

Andrea Dell'Amore

Goran Dellgren

Stefanos D. Demertzis

Michelle Demetres

Vitaly Demyanchuk

Youjun Deng

Manan Himanshu Desai

Christian Detter

Frank C. Detterbeck

Gabriele Di Giammarco

Luca Di Marco

Michele Di Mauro

Ricardo R. Dias

Vanessa Diaz-Ravetllat

Ian Diebels

Anno Diegeler

Arnaldo Dimagli

Ioannis Dimarakis

Wade Dimitri

Philipp Discher

Claudia Dittfeld

Torsten Doenst

Fabian Doerr

Fabien Doguet

Daniel-Sebastian Dohle

Pascal M. Dohmen

Chang Dong

Keyang Dong

Stefano Maria Donghi

Peter Donndorf

Augusto D'Onofrio

Vincent M. Dor

Mario Giovanni Gerardo D'Oria

Patrick Hermann Dorn

Dimitrios Dougenis

Fengmei Duan

Yves d'Udekem

Julia Dumfarth

Nandini Duraiswamy

Leonardo Duranti

Andras P. Durko

Ahmet Baris Durukan

Marek P. Ehrlich

Aschraf El-Essawi

Haytham Elgharably

Stefano Elia

Shunsuke Endo

Bastian Engel

Lars Englberger

Abdullah Erdogan

Tahir Sevval Eren

Giampiero Esposito

Anthony L. Estrera

Christian Etz

Gloria Faerber

Pierre-Emmanuel Falcoz

Wentao Fang

Abdallah Fayssoil

Mark K. Ferguson

Sandro Ferrarese

Paolo Ferrazzi

Joana Ferreira

Pia Ferrigno

Stephanie Fida

Pier Luigi Filosso

Alfonso Fiorelli

Tatjana M. Fleck

Gregory Fleming

Nikolaos Floros

Elisa Carla Fontana

Francesco Formica

Céline Forster

Sergio Nicola Forti Parri

Erik Fosse

Jose G. Fragata

Katrien Francois

Ulrich F. W. Franke

Örjan Friberg

Ikuo Fukuda

Toshihiro Fukui

Soichiro Funaki

Jozsef Furak

Michele Gallo

Carlos Galvez

Jing Gao

Yibo Gao

Jens Garbade

Rafael Garcia-Fuster

Antonio Garcia-Valentin

Gaetano D. Gargiulo

Hrvoje Gasparovic

Mario Gaudino

James William Gaynor

Amiq Gazdhar

Cengiz Gebitekin

Stephan Geidel

Hans J. Geissler

Sandro Gelsomino

Marco Gennari

Isaac George

Marc W. Gerdisch

Gino Gerosa

Behdad Gharib

Evangelos Giamarellos-Bourboulis

Alan Marc Gillinov

Daniyar Gilmanov

Evaldas Girdauskas

Walther Ivan Giron Matute

Pauline Hannah Go

Wolfgang A. Goetz

Can Gollmann-Tepeköylü

Carmen Gomar

Ana María Gómez Gago

Michel Gonzalez

Michael Gorlitzer

Dominique Gossot

Roman Gottardi

Harsha Goutham

Daniel A. Grandmougin

Stuart William Grant

Pepijn Grashuis

Juan B. Grau

Valentina Grazioli

Paulo Gregorio

Sabrina Grimaldi

Michael Grimm

William Grossi

Paul F. Gründeman

Gary L. Grunkemeier

Tomas Gudbjartsson

Dominik P. Guensch

Francesco Guerrera

Laura Chiara Guglielmetti

Robert Guidoin

Jan F. Gummert

Serdar Gunaydin

Jarmo Mikael Gunn

Jialong Guo

Rui Haddad

Peter Lukas Haldenwang

Christoph M. Haller

Yongtao Han

Claude E. Hanet

Thorsten Hanke

Henrik Jessen Hansen

Emma C. Hansson

Georgia Hardavella

Ari L. J. Harjula

Amer Harky

Majid Harmouche

Karen Harrison-Phipps

Martin Hartrumpf

Takuya Hashimoto

Wael Hassanein

Masatoshi Hata

Hoda Hatoum

Stephan Haulon

Huang-he He

David G. Healy

Michelena Hector

Claudia Heilmann

Paul Philipp Heinisch

Khosro Hekmat

Mairead Marion Hennessy

Ulrike Herberg

Israel Hernandez Ramirez

Dominik Herrmann

Samuel Heuts

Sven Hillinger

Hirofumi Hioki

Mark Hofmeyer

Bo Laksáfoss Holbek

Tomas Holubec

Haihua Hong

Katja Höpker

Xiaofeng Hou

Ilias Houda

Hung-Lung Hsu

Junjie Hu

Xiaosheng Hu

Wei Huang

Cheng-Long Huang

Christoph H. Huber

Narcis Hudorovic

Maxime Huriet

Syed Mohammad Asim Hussain

Nabil Hussein

Roy Huurman

Mohsen Ibrahim

Hitoshi Igai

Yasunori Iida

Ilkka Ilonen

Yoshito Inoue

Jessica Insolda

Selim Isbir

Norihiko Ishikawa

Ahmet Feridun Isik

Tadashi Isomura

Masahide Isowa

Keiichi Itatani

Fabio Ius

Krishna S. Iyer

Hironori Izutani

Kirolos Jacob

Marjan Jahangiri

Silje Ekroll Jahren

Oyvind Jakobsen

Anjum Jalal

William Robert Eric Jamieson

Sae K. Jang

Erik W. L. Jansen

Katarzyna Januszewska

Omar A. Jarral

Vivek Jaswal

Fabio Biscegli Jatene

Eva Maria Javier Delmo

Victor A. Jebara

Olivier J. L. Jegaden

Matija Jelenc

Anders Jeppsson

Liyan Jiang

Marcelo F. Jimenez

Federica Jiritano

Richard A. Jonas

Alberto Jorge Monteiro Dela Vega

Asher George Joseph

Sung-Ho Jung

Riyaz Kaba

Isaac Kadir

Klaus Kaier

Satoshi Kainuma

Larry R. Kaiser

Afksendiyos Kalangos

David Kalfa

Antonios Kallikourdis

Roya Kamali

Markus Kamler

Tadashi Kaname

Hiroyuki Kaneda

Emmanouil Ioannis Kapetanakis

Tevfik Kaplan

Tom R. Karl

Riyad Karmy-Jones

Alexander Emil Kaspersen

Juha Kauppi

Masashi Kawabori

Riken Kawachi

Aditya K. Kaza

Gizem Kececi Ozgur

Henrik Kehlet

Jörg Kempfert

Paula R. Keschenau

Suresh Keshavamurthy

Grigol Keshelava

Puja Gaur Khaitan

Krishna Khargi

Espeed Khoshbin

Clemens Kietaibl

Arman Kilic

Doosang Kim

Woojung Kim

Woong-Han Kim

Yong-Hee Kim

Naoyuki Kimura

Kaan Kirali

Bilal Haneef Kirmani

Hristo Kirov

Hiroto Kitahara

Yoshitaka Kitamura

Soichiro Kitamura

Robert J. M. Klautz

Patrick Klein

Theo J. Klinkenberg

Christoph Knosalla

Kinsing Ko

Jakub Maciej Kobecki

Mladen J. Kocica

Luca Koechlin

Willem Koemans

Markus Kofler

Fumitsugu Kojima

Philippe H. Kolh

Stoyan Kondov

Igor E. Konstantinov

Tomislav Kopjar

Stephan Korom

Alexander Korotkikh

Christophoros Kotoulas

Hans-Heiner Kramer

Markus Krane

George Krasopoulos

Maximilian Kreibich

Tobias Krüger

Arkadiusz Krzemiński

Bartosz Kubisa

Takashi Kunihara

Hidenori Kuno

Thomas Kuntze

Paul Kurlansky

Efrat Kurtzwald-Josefson

Jaroslaw Kuzdzal

Young-Lan Kwak

Panagiotis Grigorios Kyriazis

Mark La Meir

Sophie Lafitte

Aránzazu Lafuente Sanchis

Savvas Lampridis

Nora Lang

Emmanuel Lansac

Michael Lanuti

Stephen R. Large

Olivia Lauk

Patrick Lauwers

Robert James Leatherby

Kyongjune Benjamin Lee

Jang-Ming Lee

Eric J. Lehr

Miia L. Lehtinen

Mario Lescan

Gunda Leschber

Yijiang Li

Shanqing Li

Qiang Li

Fan Li

Peter B. Licht

Artur Lichtenberg

Faten Limaiem

Steve Livesey

Yue-Hin Loke

Chiara Lomazzi

Jose Lopez-Menendez

Roberto Lorusso

Mahmoud Loubani

Janaka Lovell

Giovanni Battista Luciani

Heyman Luckraz

Wei-Guo Ma

Jillian Madine

Balakrishnan Mahesh

Susumu Manabe

Yoshimasa Maniwa

Vito A. Mannacio

Sara Mantovani

Rita Daniela Francesca Marasco

Supreet P. Marathe

Stefano Margaritora

Mateo Marin-Cuartas

Giovanni Mariscalco

Andreas Markewitz

Luis C. Maroto

Andreas Martens

Sven Martens

Thomas Martens

Carlos Esteban Martin Lopez

Parwis Massoudy

Maria Giovanna Mastromarino

Pasquale Mastroroberto

Pankaj Mathur

Miltiadis I. Matsagkas

Hitoshi Matsuda

Haruhisa Matsuguma

Yoshiro Matsui

David Mauchley

Giulio Maurizi

Constantine Mavroudis

Antonio Mazzella

Elisa Meacci

Massimiliano Meineri

Lorenzo A. Menicanti

Ari Mennander

Olaf Mercier

Frank Merkle

Carlos M. Mery

Carlos A. Mestres

Gilles Mets

Antonio Miceli

Elisa Mikus

Milan Milojevic

Kimito Minami

Eleonora Maddalena Minerva

Martin Misfeld

Mizuki Miura

Paul Modi

Irfaan Mofeejuddy

Thomas F. Molnar

Sara Monge Blanco

Emilio Monguió

Luiz Felipe P. Moreira

Victor O. Morell

Paula Moreno

Demetrios Moris

Akimasa Morisaki

Tetsuro Morota

Victor X. Mosquera

Michael Rolf Mueller

Giacomo Murana

Takashi Murashita

Piergiorgio Muriana

Diego Alejandro Murillo Brito

John M. Murkin

Bari Murtuza

Macarena Muto

Felix Naegele

Babu Naidu

Jun Nakajima

Hiroyuki Nakajima

Apostolos Nakas

Francesco Nappi

Pradeep Narayan

Kishan K. Narine

Vinci Naruka

Filippo Naso

Rashmi Nedadur

Antonio Nenna

Eugenio Neri

Jens Neudecker

Calvin S. H. Ng

Dumbor L. Ngaage

Tom C. Nguyen

Lars Niclauss

Christoph A. Nienaber

Belen Nigro

Hiroyuki Nishi

Thilo Noack

Hiroaki Nomori

Pierluigi Novellis

Nuria M. Novoa

Gillian O'Connell

Hitoshi Ogino

Sunil Ohri

Akihiro Ohsumi

Masato Oikawa

Kenji Okada

Meinoshin Okumura

Guven Olgac

Burak Onan

Francesco Onorati

Aung Oo

Yoshihiro Oshima

Mohamed Ahmed Mohamed Osman

Tamer Ahmed Owais

Davide Pacini

Leonardo Paim

Nikolaos Thomas Panagiotopoulos

Domenico Paparella

Haralabos Parissis

Chan Hun Park

In Kyu Park

Kay-Hyun Park

Samina Park

Daniela Pasero

Gregory Pattakos

Syed Murfad Peer

Zhiyu Peng

Marco Penso

René Horsleben Petersen

Sven Peterss

Leonardo Petracca Ciavarella

Mate Petricevic

George Petrov

Gosta B. Pettersson

Stephen Pettit

Joachim O. M. Photiadis

Maximilian Pichlmaier

Ramon Pierik

Gabriele Piffaretti

Hans K. Pilegaard

Clarence Pienteu Pingpoh

Sergio Pirola

Sorin V. Pislaru

Antonios Pitsis

Nikolaus Pizanis

Bruno K. Podesser

Jan I. Poelaert

Camilla Poggi

Leo Pölzl

Cecilia Pompili

Alain Jean Poncelet

Evgenij V. Potapov

Rossella Potenza

Alberto Guido Pozzoli

Ourania Preventza

Elena Prisciandaro

Wei Chieng Pui

Salah D. Qanadli

Eduard Quintana

Mark Ragusa

Mohamed Rahouma

Mike Ralki

Karthik Vaidyanathan Ramakrishnan

Marco Ranucci

Madhuri Rao

Federico Raveglia

Sajjad Raza

Shekar L. C. Reddy

Koen D. Reesink

Hermann Reichenspurner

J Matthew Reinersman

Diana Reser

Oliver Reuthebuch

Zaccaria Ricci

Sara Ricciardi

Marc Riquet

Albert Rodríguez-Fuster

Henriette Røed-Undlien

Eric Dominic Roessner

Leonardo Roever

Luke Jonathan Rogers

Gaetano Romano

Sonia Ronchey

Antonino S. Rubino

André Rüffer

Eloisa Ruiz López

Bartosz Rylski

Michel Pompeu Sá

Diyar Saeed

Sandeep Sainathan

Tomoki Sakata

Shoji Sakiyama

Mohamed Salama

Michele Salati

Milad Samaee

Alberto Sandri

Shunji Sano

Giuseppe Santarpino

Gowthanan Santhirakumaran

Joao Santos Silva

Nishant Saran

Ulrik Sartipy

Masaaki Sato

Osamu Sato

Milton Saute

Noriyoshi Sawabata

Shahram Sayyadi

Paolo Scanagatta

Thomas Schachner

Michael Scharfschwerdt

David Schibilsky

Christian Schlensak

Jürg Schmidli

Andrej Schmidt

Christoph Schmitz

Florian Schoenhoff

Paul Schoenhogen

Richard B. Schuessler

Joerg Seeburger

Burkhardt Seifert

Yasuo Sekine

Sabina Henrika Selenius

Daniel Sellers

Paul Sergeant

Mohammad Behgam Shadmehr

Rajesh Sharma

Luisa Shaw

Jia Shi

Norihisa Shigemura

Norihiko Shiiya

Tadanaga Shimada

David Shitrit

Vivek Shrivastava

Osnat Shtraichman

Dominique Shum-Tim

Patrick Malcolm Siegel

Alan Dart Loon Sihoe

John Simpson

Pranava Sinha

Thanos Sioris

Mark Slaughter

Freyja Maria Smolle-Jüttner

Laura Socci

Sebastian-Patrick Sommer

Howard Song

Wenpeng Song

Makoto Sonobe

Lorenzo Spaggiari

Sabine Spänig

Mila Stajevic

Steven Aleksandar Stamenkovic

Olaf Stanger

Christoph Thomas Starck

Alessandro Stefani

Marie-Elisabeth Stelzmueller

Serban C. Stoica

Seher Susam

Piotr Suwalski

Staffan Svenmarker

Ben M. Swinkels

Simo Syrjälä

Gabor Szabo

Kazuyoshi Takagi

Tamaki Takano

Aikaterini Christina Tampaki

Kunyue Tan

Wilco Tanis

Maurizio Taramasso

Vakhtang Tchantchaleishvili

Adele Tessitore

Stefan Thelin

Matthieu Thumerel

Kin-Hoi Thung

Fei Tian

Hongtao Tie

Tomasz Timek

Alper Toker

Anton Tomsic

Aybala Tongut

Francesca Toto

Ioannis K. Toumpoulis

Nikolay O. Travin

Hendrik Treede

Santi Trimarchi

Konstantinos Tsagakis

Nikolaos Tsilimparis

Akif Turna

Naomichi Uchida

Murat Ugurlucan

Jarle Vaage

Caroline Van De Heyning

Caroline Van De Wauwer

Charlotte Van Edom

Arnaud Van Linden

Karel M. Van Praet

Dirk E. M. Van Raemdonck

Paul Van Schil

Korneel Vandewiele

Panos Vardas

Gonzalo Varela

Alejandro Vazquez

Kevin M. Veen

Tiago R. Velho

Prem Sundar Venugopal

Ad Verhagen

Giulia Veronesi

Vladimiro L. Vida

Vibeke Videm

Emmanuel Villa

Hunaid A. Vohra

Jan Vojacek

Luca Voltolini

Konstantin Von Aspern

Kerem M. Vural

Andrew Waberski

Alexander Wahba

Isaac Wamala

Zhenping Wan

Chunsheng Wang

Gregor Warnecke

Kenneth James Warring-Davies

Yui Watanabe

Atsushi Watanabe

Stefan B. Watzka

Alberto Weber

Barbara Weckler

Francis Wells

Luca Paolo Weltert

Alexander Weymann

Dominik Wiedemann

Randolph H. L. Wong

Hao Wu

Ming-Ho Wu

Dong Xie

Hugo Xochitemol Herrera

Gaosi Xu

Yoshitaka Yamane

Mohammed Yasin

Can Yerebakan

Hu Yijin

Murat Yildiz

Bedrettin Yildizeli

Kanhua Yin

Nizar Yonan

Jia Yu

Joseph Zacharias

Sergey Zaets

Ralf Zahn

Edoardo Zancanaro

Konstantinos Zannis

David Zapata

Francesco Zaraca

Yan Zhang

Yang Zhang

Alicja Zientara

Charalambos Zisis

Andrea Zuin

Yunxia Zuo

